# Effects of Message Frames and Sources in TikTok Videos for Youth Vaping Cessation: Emotions and Perceived Message Effectiveness as Mediating Mechanisms

**DOI:** 10.1016/j.jadohealth.2024.08.013

**Published:** 2024-10-04

**Authors:** Jiaxi Wu, Jessica L. Fetterman, Jennifer Cornacchione Ross, Traci Hong

**Affiliations:** a Annenberg School for Communication, University of Pennsylvania, Philadelphia, Pennsylvania; b Evans Department of Medicine and Whitaker Cardiovascular Institute, Boston University, Chobanian & Avedisian School of Medicine, Boston, Massachusetts; c Department of Health Law, Policy & Management, Boston University School of Public Health, Boston, Massachusetts; d College of Communication, Boston University, Boston, Massachusetts

**Keywords:** TikTok, Vaping cessation, e-cigarette, Youth, Health campaign, Social media

## Abstract

**Purpose:**

The objective of this study was to evaluate the effect of message frames (gain vs. loss) and sources (formal expert: a health-care professional vs. informal expert: an individual who successfully quit vaping) on the persuasiveness of TikTok videos among youth who currently use e-cigarettes. Additionally, the study examined how emotional responses and perceived message effectiveness (PME) mediate the framing effect on youth intention to quit vaping.

**Methods:**

A 2 (gain frame vs. loss frame) × 2 (formal expert vs. informal expert) factorial design experiment was conducted with 378 youth aged 13 to 18 who currently use e-cigarettes. The study measured participant’s emotional responses, PME of the messages, and intention to quit vaping after the video exposure.

**Results:**

Messages from a formal expert resulted in stronger intention to quit vaping compared to messages from an informal expert. Gain-framed messages were associated with higher PME when delivered by an informal expert, whereas loss-framed messages showed stronger effects on PME from a formal expert. Positive emotional responses and increased PME mediated the relationship between gain-framed messages and youth intention to quit vaping.

**Discussion:**

TikTok could serve as an effective tool for formal experts to promote vaping cessation among youth who use e-cigarettes. Additionally, the findings suggest that gain frames may be more influential than loss frames in promoting vaping cessation among youth, by eliciting positive emotional responses from the audience. The differential impact of message frames depending on source type indicates a nuanced interaction between content and messenger.

The use of electronic cigarettes, or vaping, has seen a significant rise among youth worldwide in recent decades. A study conducted across 47 countries from 2015 to 2018 found that approximately one in 12 youth (8.6%), reported vaping in the past 30 days [1]. In the United States, over 2 millions of middle and high school students reported past 30-day use of e-cigarettes, with 25% of current users vaping daily in 2023 [2]. Despite the high prevalence of youth e-cigarette use, there has been limited focus on vaping cessation campaigns for this population.

Short video content, typically do not exceed one or a few minutes, has gained immense popularity across social media platforms. TikTok, the most popular short video app, has amassed over 4.1 billion global downloads [3]. Riding the wave of TikTok’s success, YouTube and Instagram have both introduced short video features, namely YouTube Shorts and Instagram Reels. Short videos present a promising avenue for disseminating health information to youth [4]. On average, youth spend approximately 4.8 hours per day on social media, with around 7-in-10 reporting daily visits to video-sharing platforms [5]. Research indicates that 87% of vaping-related TikTok videos are either positive or neutral, with 30% of videos being promotional content for e-cigarettes [6]. To counter the widespread promotion of e-cigarettes on TikTok and engage the younger population, research is needed to understand effective content features and sources tailored specifically for short video anti-vaping campaigns.

## Literature Review

### Desired persuasive outcomes of vaping cessation short videos

Enhancing perceived message effectiveness (PME) and promoting intention to quit vaping are desirable outcomes of vaping cessation short videos. PME refers to individuals’ perceptions of how effective a health message is in bringing about behavior change, which is a widely employed measure to assess the direct outcomes of exposure to a persuasive health message [7]. Behavioral intention, on the other hand, is the most immediate and important predictor of behavior according to the Theory of Plan Behavior (TPB) [8]. The TPB posits that an individual’s behavior is determined by their intentions to perform that behavior. Extensive empirical evidence underscores the efficacy of TPB in predicting health-related behaviors, including tobacco use [9].

### Prospect theory and message framing to reduce youth vaping

Prospect theory suggests that individuals are more inclined to take risks when faced with messages emphasizing potential benefits (gain framing), and more likely to avoid risks when encountering messages highlighting potential negative consequences (loss framing) [10]. In health communication, researchers propose that gain framing better promote preventive behaviors such tobacco cessation, while loss framing is more effective for risk-related detection behaviors, like cancer screenings [11].

In the context of tobacco use, previous research on the framing effect has predominantly centered on smoking cessation messages aimed at adults, with one meta-analysis highlighting an advantage for the gain framing [12]. However, recent literature indicates that loss framing is generally more effective than gain framing in discouraging vaping and promoting smoking cessation among youth [13–16]. For instance, loss-framed vaping prevention messages increased risk perceptions and reduced vaping intentions compared to gain-framed messages [14]. Loss-framed vaping prevention messages also elicited stronger negative emotions [13] and were preferred by youth [15]. In smoking cessation research, loss-framed messages were similarly associated with more positive attitudes toward quitting smoking than gain-framed messages among youth [16].

Nevertheless, a recent systematic review reveals that previous research on message framing has predominantly focused on vaping prevention, with no studies investigating message framing for youth vaping cessation [17]. This study seeks to address this gap by investigating the framing effects of vaping cessation TikTok videos on youth. Based on the accumulated evidence indicating the effectiveness of loss frames in reducing youth tobacco use [13–16], we propose the following hypothesis:

*H1*. Loss-framed vaping cessation videos are associated with increased PME (H1a) and enhanced intention to quit vaping (H1b) compared to gain-framed videos among youth using e-cigarettes.

### Formal and informal expert sources in health communication

High-credibility message sources, such as health-care professionals with specialized knowledge, are more persuasive than low-credibility sources in encouraging changes in attitudes and behaviors [18]. Health-care professionals have utilized TikTok to share public health information on diverse topics [19], though research has not yet examined how youth respond to vaping cessation videos from health-care professionals.

Recent research has recognized the persuasive effect of experiential expertise, which stems from personal experience rather than formal qualifications [20]. Individuals often turn to the internet to find others with shared health experiences for health information, making experiential credibility particularly influential online [21]. For instance, readers of a personal HIV blog showed more positive attitudes and increased self-efficacy about condom use compared to visitors of an official health website [22].

While no research has specifically examined the effects of formal expert source versus informal expert source on youth intention to quit vaping, a study on youth smoking cessation reported that youth preferred smoking cessation videos delivered by successful youth quitters, who hold personal experience of quitting than health-care professionals such as physicians [16]. Based on literature highlighting the benefits of informal expert over formal expert in online health information evaluation [22] and youth preference for experiential expertise in smoking cessation messages [16], we proposed the following hypothesis:

*H2*. Vaping cessation videos from informal experts are associated with increased PME (H2a) and enhanced intention to quit vaping (H1b) compared to videos from formal experts among youth using e-cigarettes.

### Interaction of message frames and sources

The interaction between frames and sources on message effects has received relatively limited attention. Research suggests that gain framing is more effective for audiences who perceive a similarity with the character in the message [23]. Loss-framed messages delivered by formal experts like doctors may have a greater impact due to the authoritative stance of experts, underscoring the seriousness of inaction [24]. For example, patients were more likely to pursue cancer treatment when the doctor employed loss-framed language [25]. Therefore, informal experts, who share similarities in vaping use, can be a powerful model for behavior change when using gain-framed messages, whereas formal experts are more effective with loss-framed messages emphasizing the consequences of not quitting. We proposed the following hypothesis:

H3. Message frames and sources interact to influence PME (H3a) and intention to quit vaping (H3b) among youth, with gain-framed vaping cessation videos being more effective when conveyed by informal expert sources and loss-framed videos being more effective when delivered by formal expert sources.

### The mediating effect of emotional responses in framing effects

Another objective of this study is to examine the mechanism through which the framing effect occurs in the context of messaging for youth vaping cessation. The emotions-as-frames model posits that emotions shape information processing and decision-making, with loss-framed messages typically inducing negative emotions and gain-framed messages eliciting positive emotions [26]. Furthermore, the emotions-as-frames model argues that the persuasive effect of gain-framed messages depends on the intensity of positive emotions induced, while the influence of loss-framed messages is contingent upon the intensity of negative emotions [26]. A previous meta-analysis suggests that PME of tobacco education campaigns predicted quit intentions [27]. Therefore, in this mediation analysis, we treated PME as a secondary mediator to the outcome variable of youth intention to quit vaping.

H4: Positive emotional responses and PME mediate the relationship between gain frame and persuasive outcomes.

H5: Negative emotional responses and PME mediate the relationship between loss frame and persuasive outcomes.

In summary, this study investigates how message frames and sources in TikTok videos affect PME and youth intention to quit vaping. Additionally, we explore the mediating role of emotional responses and PME in the effects of framed TikTok videos on youths’ intention to quit.

## Methods

### Study design

A 2 (frame: gain vs. loss) × 2 (message source: health-care professional vs. individual who quit vaping) online experimental study was performed on Qualtrics from May 11 to May 25, 2023. The study protocol was approved by the Institutional Review Board of the first authors’ institute. In consideration of the sensitive nature of disclosing vaping status among participants under the age of 18, the requirement for parental consent was waived. Instead, direct assent was sought and obtained from each youth participant.

### Participants

Participants aged 13-18 were recruited using Qualtrics Research Services. To be eligible for inclusion in the study, participants were required to self-report current use of e-cigarettes within the past 30 days.

### Procedure

Participants first responded to questions concerning their e-cigarette use behaviors. Respondents who did not use e-cigarettes in the past 30 days were excluded from the survey. Subsequently, using Qualtrics’ built-in randomizer, participants were randomly assigned to view one of four short TikTok videos centered around quitting e-cigarettes. After viewing the video, participants were asked a series of questions regarding persuasive outcome variables and provided demographic information.

### Stimuli

For the study, 4 TikTok videos were created, each edited from an existing #quitvaping video to maintain authenticity, featuring a vape being submerged in water. The person’s face in the video was intentionally concealed, with only their hand visible. The original audio was replaced with AI-generated voiceovers, and the script, based on previous framing research on youth vaping [14,15], was displayed as captions. The scripts, screenshots, and QR codes for all videos are detailed in Table 1. The videos range from 24 to 27 seconds.

The TikTok videos discuss the physical health, addiction, and mental health consequences of e-cigarette use. Video framing was manipulated to discuss the health consequences of vaping (loss framing) versus the benefits of quitting (gain framing). Individuals who successfully quit vaping are considered informal experts, based on their firsthand experiences in overcoming the challenges related to e-cigarette cessation. In contrast, formal experts in this study were operationalized as health-care professionals. To manipulate the message source, the videos in the informal expert message source condition explicitly introduced oneself as someone who successfully quit vaping and used a handle “@VapeNoMore2023”, while the video in the formal expert condition introduced oneself as a doctor and used ““@DrRobJohnson.” A pilot study confirmed effective manipulation of message frames and sources, with detailed results in the supplementary materials.

### Measurement

#### Perceived message effectiveness (PME).

PME was measured by 3 questions on a Likert-scale ranging from 1 (not at all) to 5 (a great deal): 1) “How much does this video make you worry about the potential effects of vaping on your health?” 2) “How much does this video make you believe that vaping is a bad idea?” 3) “How much does this video discourage you from vaping?” [28]. Composite scores were derived for PME by computing the mean of the 3 items.

#### Intention to quit vaping.

This single-item assessment [29], asks participants to indicate on a scale from 0 (no thought of quitting) to 10 (taking action to quit such cutting down, enrolling in a program) how intended they are to quit e-cigarette use.

#### Positive emotional responses.

After viewing the TikTok video, participants reported how they felt during the video exposure. The items included hopeful, determined, and inspired for positive emotional responses [14]. Participants rated each item on a 5-point Likert-scale from “not at all” to “a great deal.”

#### Negative emotional responses.

Similarly, 3 items including worried, afraid, and uneasy were used for measuring negative emotional responses [14]. Participants also rated each item on a 5-point scale from “not at all” to “a great deal.”

#### Control variables.

Building on prior research highlighting the potential moderating effects in message framing, factors including gender [30], vaping dependence [31], baseline quitting intention (i.e., quitting stages) [32], quitting outcome expectancy (i.e., vaping risk perceptions) [30], and issue involvement in vaping cessation [33] were controlled for in the current study.

#### Gender.

Youth self-reported demographics were adjusted for in this study, which include options of woman, man, nonbinary, and prefer not to tell.

#### Vaping dependence.

Vaping dependence was measured with the 4-item Patient-Reported Outcomes Measurement Information System Nicotine Dependence Item [34].

#### Baseline quitting intention (i.e., quitting stages).

Participants were asked about their intentions to quit vaping within different time frames: the next week, month, 6 months, 12 months, or if they did not intend to quit within the next 12 months. A continuous variable ranging from 1 to 5 was subsequently created to indicate the baseline quitting intention (i.e., quitting stages) among the participants.

#### Quitting outcome expectancy.

The scale included 22 items [35], and responses are scored on a 5-point Likert scale with items ranging from 0 (very unlikely) to 5 (very likely). Example questions included “I will feel a sense of achievement” and “I will be less able to concentrate.” Positive outcome expectancy items were reverse coded to keep consistency of the scale. Higher total scores on the measurement indicate greater negative expectancies from abstaining from e-cigarettes.

#### Issue involvement of quitting vaping.

Following previous research [33], youth were asked the question “I think quitting e-cigarettes is.” Responses were measured with a 5-point semantical differential scale with 3 statements: unimportant–important, irrelevant–relevant, means nothing to me–means a lot to me.

### Statistical analysis

H1 to H3 were analyzed with analysis of covariance (ANCOVA). Experimental factors (i.e., message frames and message sources) and their interaction were submitted to ANOCOVA models as independent variables. Outcome variables of the ANOCOVA models were PME and intention to quitting vaping.

To investigate H4 and H5, we performed a mediation analysis. Message framing was the predictor, while positive emotions and negative emotions served as 2 parallel mediators sequentially preceding a third mediator, PME, and finally, the dependent variable, which is the intention to quit vaping. Hayes’ PROCESS macro (PROCESS Model 80) was used with bootstrap confidence intervals for indirect effects between predictors and outcome variables through mediators.

Both the ANCOVA and the mediation analyses were adjusted for participants’ gender, vaping dependence, baseline quitting intention, quitting outcome expectancy, and issue involvement of quitting vaping.

## Results

A manipulation check confirmed successful manipulation of both the message source and frame in the study, with detailed information provided in the supplementary materials.

378 youth aged 13 to 18 who are current e-cigarette users participated in this study. Participants were randomly assigned as follows: 98 to gain frame-informal expert, 94 to gain frame-formal expert, 94 to loss frame-informal expert, and 92 to loss frame-formal expert conditions. Most participants identified as White/Caucasian (66%), followed by Black/African American (14%), multiracial (10%), Asian (4%), American Indian or Alaska Native (3%), and Native Hawaiian or Other Pacific Islander (3%). Regarding gender, 66.9% identified as female, 22.5% as male, 5.3% as nonbinary, and 5.3% preferred not to disclose their gender. The average age of participants was 17 (SD = 1.1). Supplemental Table 1 presents the descriptive results for the study’s mediators, outcome and control variables.

### Hypothesis and research question testing

H1 predicted that the loss framing is associated with increased PME (H1a) and enhanced intention to quit vaping (H1b). The analysis indicated no significant main effect of framing. H1a and H1b were rejected.

H2 proposed that the informal message source evokes increased PME (H2a) and enhanced intention to quit vaping (H2b). The ANCOVA analysis suggests that formal expert sources led to higher intention to quit vaping compared to informal expert sources (*p* = .04, $p/\eta_{p}^{2}=0.012$). Neither H2a nor H2b were supported, with H2b showing an unexpected inverse relationship between message sources and intention to quit vaping. Table 2 summarizes the main effects of message frames and sources.

**Figure 1.**
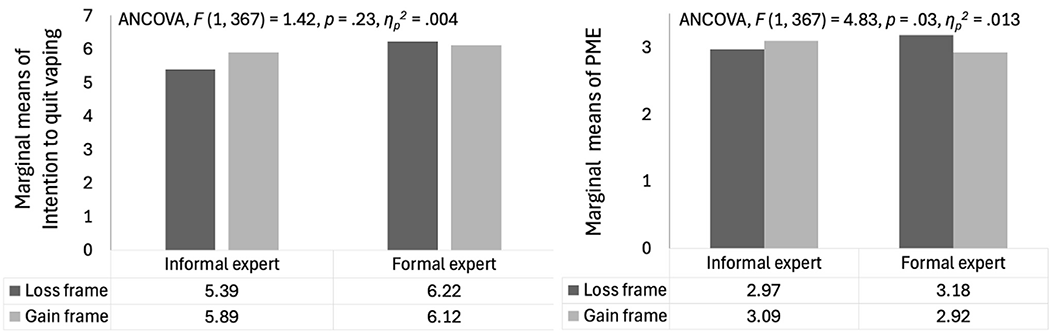
Interaction effects between message frames and sources on PME and intention to quit vaping. PME = perceived message effectiveness. **Note**. Both models were adjusted for participants’ gender, vaping dependence, baseline quitting intention, quitting outcome expectancy, and issue involvement of quitting vaping.

H3 predicted that gain-framed vaping cessation videos are more effective with an informal expert source, whereas loss-framed videos are more effective with a formal expert source. This interaction was found significant for PME (*p* = .03, $p/\eta_{p}^{2}=.013$). For video portrayed a formal expert, the loss frame elicited higher PME than gain frame. On the contrary, for video portrayed an informal expert, the gain frame elicited higher PME than loss frame. Therefore, H3a was supported. The interaction between frames and sources on intention to quit vaping was not significant (*p* = .23, $p/\eta_{p}^{2}=.004$). H3b was not supported. The interaction effects of message framing and sources are plotted in Figure 1.

H4 proposed that positive emotional responses and PME mediate the relationship between gain framing and youth intention to quit vaping. Results from the serial mediation analysis showed that the indirect effects of gain framing on intention to quit vaping through positive emotional responses and PME were significant (B = 0.04, Bootstrap 95% CI: 0.001, 0.09). Therefore, H4 was supported. Detailed direct and indirect effects of the mediation analysis are reported in Table 3.

**Figure 2.**
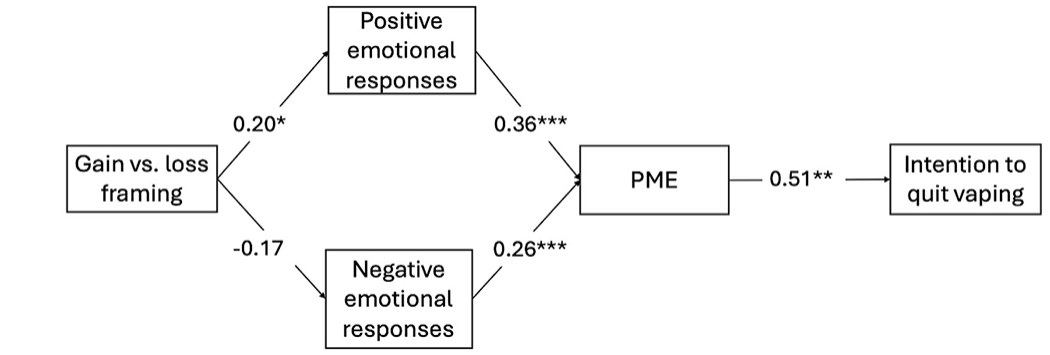
Diagram of unstandardized regression coefficients in the serial mediation model. **Note**. Results of the mediation model of message framing on intention to quit vaping through positive and negative emotional responses and PME. Unstandardized regression coefficients are reported for each path. The model was adjusted for gender, vaping dependence, baseline quitting intention, quitting outcome expectancy, and issue involvement in quitting vaping. Estimates were calculated using the PROCESS macro (model 80). * *p* ≤ .05, ** *p* ≤ .01, *** *p* ≤ .001.

Lastly, H5 suggested that negative emotional responses mediate the relationship between loss framing and youth intention to quit vaping. However, the indirect effects of loss framing on intention to quit vaping through negative emotional responses and PME (B = −0.02, Bootstrap 95% CI: −0.06, 0.003) was not significant. Thus, H5 was not supported. Figure 2 presents the unstandardized regression coefficients in the examined mediation models.

## Discussion

This study examined the main and interaction effects of message frames and sources in TikTok videos on persuasive outcomes, specifically PME and the intention to quit vaping among youth who currently use e-cigarettes. We also investigated the role of emotions and PEM as mediators in the relationship between message frames and intention to quit vaping.

Our findings highlight the effectiveness of using gain-framed videos to promote youth vaping cessation by eliciting positive emotions. Our findings align with prospect theory and existing literature supporting gain framing’s benefits in smoking cessation among adults [10–12]. However, our results challenge previous studies suggesting loss framing is more effective in deterring vaping and promoting smoking cessation among youth [13–16]. One possible explanation is that prior research on framing effects in youth vaping has primarily focused on prevention rather than cessation [13–15]. Since youths’ preferences for framing in vaping prevention messages vary based on individual characteristics, including vaping status [15], findings from prevention studies may not apply to the context of cessation.

Furthermore, unlike prior research using 11-minute smoking cessation videos [16], our study utilized TikTok short videos lasting less than half a minute. The effectiveness of gain framing and positive emotions in our study may be attributed to heuristic processing of social media short videos [36], where individuals tend to favor positive information consistent with the hedonic principle [37]. Our findings emphasize the need for additional research on how various message features beyond framing impact audience emotional responses to short video health campaigns on social media.

Contrary to our hypothesis, our research indicated that formal experts were more persuasive in motivating youth towards quitting than informal experts. This may be because the videos emphasized reasons to quit rather than personal quitting stories or tips for quitting. Construal level theory posits that psychological distance influences how messages are interpreted [38]. Construal level theory considers several dimensions of psychological distance including temporal (time closeness), spatial (physical distance), social (interpersonal distance), and hypothetical (likelihood of events). Youth may see individuals with similar vaping experiences as socially closer than formal experts, as social vaping has become a bonding activity among youth [39]. Therefore, informal experts, being perceived as more proximal than formal experts, may not be as effective in conveying the abstract concept of why to quit as more concrete ideas of how to quit. Research suggest that matching the construal level across message components such as topics, sources, and information processing style will lead to more persuasive effects [40]. Hence, future studies should explore how the source and content of messages (such as reasons to quit, personal experiences, and strategies for overcoming barriers) affect the message persuasiveness.

Our study found that gain-framed messages were more effective when delivered by informal experts, and loss-framed messages resonated more when presented by formal experts. The findings suggested that the persuasiveness of message framing is influenced by the message source. Specifically, in promoting vaping cessation among youth, messages about benefits (gain-framed) from peers or informal sources may be more relatable [23], while formal experts’ authority lends weight to the consequences of vaping (loss-framed) [24].

Our study has limitations. Although the manipulation check confirmed that participants successfully identified the health-care professional message source in the formal expert condition, the lack of the presence of the face of a real health-care professional and the use of AI-generated voiceovers may diminish the authenticity and influence of the formal expert videos. Future research with adequate resources should also explore collaborations with real health-care professionals or actors to enhance the authenticity of the videos. Our study exposed participants to a single TikTok video in an experimental setting. Investigating the long-term effects of exposure to vaping cessation TikTok videos and conducting observational studies to explore the relationship between exposure and cessation behaviors could provide additional insights for future short video campaigns aimed at promoting vaping cessation.

Theoretically, this study enriches the literature by examining how message frames and sources influence the impact of vaping cessation TikTok videos among youth. Additionally, our findings underscore the pivotal role of emotions in fostering behavioral change through short video social media platforms. Practically, our findings suggest that short video platforms offer a promising avenue for formal experts, such as health-care professionals, to disseminate health information and foster positive behavioral changes.

## Supplementary Material

MMC1

## Figures and Tables

**Table 1:** Transcriptions, screenshots, and QR codes of Video Stimuli

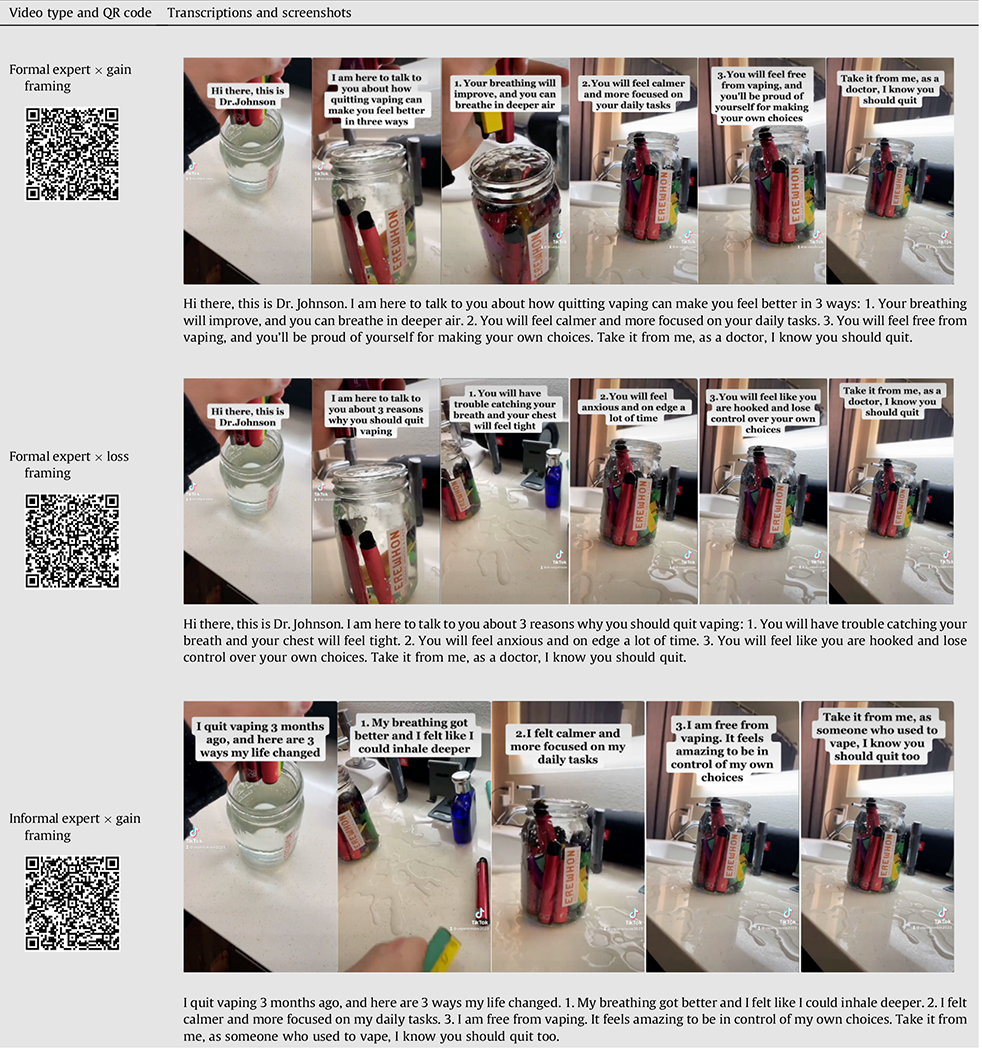
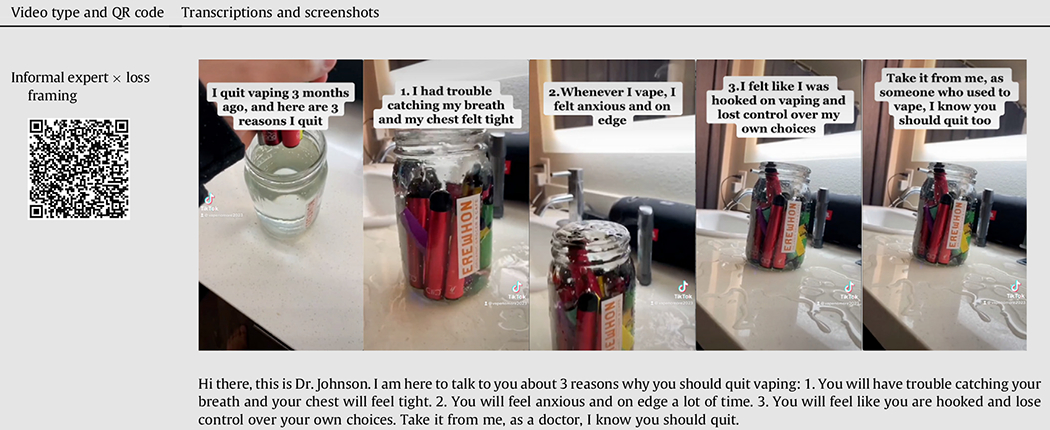

**Table 2:** Descriptive and main effects of message frames and message source type

	Loss M (SD)	Gain M (SD)	$p/\eta_{p}^{2}$	Informal expert M (SD)	Formal expert M (SD)	$p/\eta_{p}^{2}$
PME	3.08 (0.06)	3.01 (0.06)	0.42 (0.002)	3.03 (0.06)	3.05 (0.06)	0.82 (0.000)
Intention to quit vaping	5.81 (0.18)	6.00 (0.18)	0.45 (0.002)	5.64 (0.17)	6.16 (0.18)	**0.04 (0.012)**

Numbers for each condition are means and standard errors, adjusting for gender, vaping dependence, baseline quitting intention, quitting outcome expectancy, and issue involvement of quitting vaping. Bold text indicates statistically significant results.

PME = perceived message effectiveness; SD = standard deviation.

**Table 3:** Mediation model with model summary, direct effect, and indirect effect

Predictors	R^2^	Coef.	SE	*t*	*p*	95% bias-corrected CI
Outcome: positive emotions	.27				< .001	
Constant		1.08	.39	2.80	.01	0.32–1.84
Gain frame		**.20**	**.10**	**2.05**	**.04**	**0.01–0.39**
Gender (reference group: female)						
Male		−0.16	.12	−1.36	.17	−0.39 to 0.07
Nonbinary		−0.24	.22	−1.10	.27	−0.67 to 0.19
Prefer not to tell		.26	.22	1.18	.24	−1.17 to 0.69
Vaping dependence		.08	.06	1.36	.18	−0.04 to 0.19
Baseline quitting intention		**.19**	**.04**	**5.31**	**<.001**	**0.12–0.26**
Quitting outcome expectancy		−0.04	.09	−0.47	.64	−0.21 to 0.13
Issue involvement of quitting vaping		**.37**	**.04**	**8.36**	**<.001**	**0.28–0.45**
Outcome: negative emotions	.17				<.001	
Constant		.31	.36	.85	.40	−0.40 to 1.01
Gain frame		−0.17	.11	−1.61	.11	−0.38 to 0.04
Gender (reference group: female)						
Male		−0.21	.13	−1.67	.10	−0.46 to 0.04
Nonbinary		−0.06	.24	−0.27	.79	−0.53 to 0.41
Prefer not to tell		.26	.24	1.09	.27	−0.21 to 0.72
Vaping dependence		.05	.06	.76	.45	−0.08 to 0.17
Baseline quitting intention		**.19**	**.04**	**4.92**	**<.001**	**0.12–0.27**
Quitting outcome expectancy		**.25**	**.10**	**2.58**	** .01**	**0.06—0.43**
Issue involvement of quitting vaping		**.25**	**.05**	**5.29**	**<.001**	**0.16–0.35**
Outcome: PME	.60				<.001	
Constant		.34	.23	1.46	.14	−0.12 to 0.80
Gain frame		−0.09	.07	−1.35	.18	−0.23 to 0.04
Positive emotional responses		**.36**	**.04**	**8.96**	**<.001**	**0.28–0.44**
Negative emotional responses		**.26**	**.04**	**7.12**	**<.001**	**0.19–0.33**
Gender (reference group: female)						
Male		−0.11	.08	−1.29	.20	−0.27 to 0.06
Nonbinary		−0.13	.16	−0.84	.40	−0.44 to 0.18
Prefer not to tell		.02	.15	.14	.89	−0.28 to 0.33
Vaping dependence		.03	.04	.70	.49	−0.5 to 0.11
Baseline quitting intention		.01	.03	.34	.73	−0.04 to 0.06
Quitting outcome expectancy		−0.06	.06	−1.00	.32	−0.19 to 0.06
Issue involvement of quitting vaping		**.29**	**.03**	**8.52**	**<.001**	**0.22–0.36**
Outcome: intention to quit vaping	.52				<.001	
Constant		−1.09	.75	−1.44	.15	−2.56 to 0.40
Gain frame		.14	.22	.65	.52	−0.29 to 0.58
Positive emotional responses		**.75**	**.14**	**5.32**	**<.001**	**0.47–1.03**
Negative emotional responses		**.39**	**.13**	**3.10**	**.002**	**0.14–0.64**
PME		**.51**	**.17**	**3.03**	**.003**	**0.18–0.83**
Gender (reference group: female)						
Male		**.66**	**.27**	**2.47**	**.01**	**0.13–1.18**
Nonbinary		**1.51**	**.50**	**3.04**	**.003**	**0.53–2.49**
Prefer not to tell		−0.25	.50	−0.51	.61	−1.22 to 0.72
Vaping dependence		−0.01	.13	−0.06	.95	−0.27 to 0.25
Baseline quitting intention		**.42**	**.09**	**4.92**	**<.001**	**0.25–0.59**
Quitting outcome expectancy		−0.39	.20	−1.93	.054	−0.78 to 0.01
Issue involvement of quitting vaping		**.50**	**.12**	**4.22**	**<.001**	**0.27–0.74**
Indirect effects						
Total indirect effect		.05	.13			−0.22 to 0.31
Gain framing → positive emotions → intention to quit vaping		**.15**	**.08**			**0.01–0.32**
Gain framing → negative emotions → intention to quit vaping		−0.07	.05			−0.17 to 0.01
Gain framing → PME → intention to quit vaping		−0.05	.04			−0.15 to 0.02
Gain framing → positive emotional responses → PME → intention to quit vaping		**0.04**	**.03**			**0.001–0.09**
Gain framing → negative emotional responses → PME → intention to quit vaping		−0.02	.02			−0.06 to 0.003
Direct effect of framing on intention to quit		.14	.22	.65	.52	−0.29 to 0.58
Total effect		.19	0.25	.77	.44	−0.30 to 0.69

Bold text indicates statistically significant results.

CI = confidence interval; Coef = unstandardized coefficients; PME = perceived message effectiveness; SE = standard error of beta coefficients.
