# Supplementary material for: Effects of Message Frames and Sources in TikTok Videos for Youth Vaping Cessation: Emotions and Perceived Message Effectiveness as Mediating Mechanisms
Source: J Adolesc Health. Author manuscript; Available in PMC 2025 Jan 1. (PMC11655248; doi:10.1016/j.jadohealth.2024.08.013)
Supplement: MMC1 [file NIHMS2027688-supplement-MMC1.docx]

**Effects of Message Frames and Sources in TikTok Videos for Youth Vaping Cessation: Emotions and Perceived Message Effectiveness as Mediating Mechanism**

Supplementary Materials

Table of Contents

[Power Analysis 2](#_Toc169728473)

[Pilot Test of Variable Manipulation 2](#_Toc169728474)

[Manipulation Check 3](#_Toc169728475)

[Supplemental Table 1. Outcome, mediating and control variables in the study 4](#_Toc169728476)

# Power Analysis

A preliminary power analysis was carried out using G*Power to determine the appropriate sample size for this study. Based on the small effect size observed in previous meta-analyses on gain and loss framing effects (Gallagher & Updegraff, 2012; O’Keefe & Jensen, 2006; O'Keefe & Nan, 2012), a desired statistical power level of 95% with an α value of .05, a sample size of 378 was determined to be adequate.

# Pilot Test of Variable Manipulation

Before implementing the experiment with the 378-youth sample recruited from Qualtrics, a pilot test was conducted among undergraduate students from a major university. The purpose of the pilot test was to assess the effectiveness of the frames and source manipulations in the video stimuli. A total of 127 undergraduate students voluntarily participated in the pilot study and received extra course credit for their participation. Participants were randomly assigned to one of four video conditions on the Qualtrics survey platform. After viewing the videos, participants were asked to indicate whether the video was presented by a doctor and whether it was from someone who successfully quit vaping. The chi-square analyses revealed significant findings for source identification in both the informal expert condition, *χ²* (1) = 70.99, *p* < .001, and the formal expert condition, *χ²* (1) = 92.48, *p* < .001.

To evaluate the manipulation of message frames, participants were asked to assess the overall tone of the video as predominantly negative or positive (Steward et al., 2003). Chi-square analysis results showed that participants in the gain-frame condition perceived the overall tone of the video as more likely to be positive compared to those in the loss-frame condition, *χ²* (1) = 55.19, *p* < .001. Additionally, participants responded to a semantic differential question assessing the emphasis of the videos, including whether the videos emphasized the risks of vaping/benefits of quitting, the loss of vaping/gain of quitting, and the negative aspects of vaping/positive aspects of quitting (Shen & Dillard, 2009; Wong et al., 2013). Independent samples t-tests indicated significant differences between the participants in gain-frame and loss-frame conditions for perceived emphasis on the benefits of quitting (*t* (125) = 8.71, *p* < .001), perceived gain from quitting (*t* (125) = 3.58, *p* < .001), and perception of positive aspects of quitting (*t* (125) = 7.09, *p* < .001). Both analyses suggest successful manipulation of message frames of the video stimuli.

# Manipulation Check

Consistent with the pilot study, participants were asked two questions to assess the manipulation of message sources. The first question inquired whether the video was presented by a doctor, while the second question asked whether the video was posted by someone who successfully quit vaping. Chi-square analyses yielded significant results for source identification in both the informal expert condition, χ² (1) = 267.55, p < .001, and the formal expert condition, χ² (1) = 115.72, p < .001.

To check the manipulation of massage frames, participants were asked if the video has an overall tone of positive or negative. The chi-square analysis revealed that participants in the gain-framed condition were more likely to perceive the overall tone of the video as positive compared to those in the loss-framed condition, χ² (1) = 48.14, p < .001. Additionally, independent sample t-tests on a semantic differential scale showed that participants in the gain-framed condition rated the gain frame significantly higher in terms of emphasizing the benefits of quitting (t (376) = 9.16, p < .001), perceived gain from quitting (t (376) = 4.48, p < .001), and positive aspects of quitting (t (376) = 9.73, p < .001) compared to the loss-framed condition. These results indicate the successful manipulation of both the message source and frame in the study.

# Supplemental Table 1. Descriptive results of outcome, mediating and control variables

| **Construct** | **Mean** | **Standard Deviation** | **Cronbach's α** |
| --- | --- | --- | --- |
| Perceived Message Effectiveness (PME) | 3.04 | 1.02 | 0.84 |
| Intention to Quit Vaping | 5.90 | 2.98 | NA |
| Positive Emotional Responses | 2.96 | 1.07 | 0.80 |
| Negative Emotional Responses | 2.61 | 1.09 | 0.82 |
| Vaping dependence | 3.01 | 0.95 | 0.76 |
| Baseline Quitting Intention (i.e., Quitting Stages) | 2.86 | 1.36 | NA |
| Quitting Outcome Expectancy | 3.83 | 1.12 | 0.85 |
| Issue Involvement of Quitting Vaping | 3.07 | 0.63 | 0.83 |
